# Bad Cooking

**Published:** 1876-06

**Authors:** 


### *BAD COOKING.*

Vast quantities of provisions are not only wasted, but made positively injurious and even dangerous to health and ultimately to life itself, by bad cooking. The American people "bolt" more crude, improperly cooked and indigestible food every day than the French or English do in a year. Hence, we are a nation of dyspeptics. There are not ten hotels or restaurants in the City of New York, managed by Americans, where a wholesome meal can be procured, and as to their coffee, we should much prefer a dose of jalap to the article usually served.

American people do not "live to eat so much as they eat to live." So long as food can be hacked into shape to be swallowed, it seems to matter very little of what it is composed or how it is prepared. This is all wrong; more reason and intelligence must be infused into the culinary department of every home, to prevent its becoming a private hospital.

### POOR ECONOMY.

It is customary for farmers to sell the best of their farm produce, and keep the poorest to be consumed at home. This is the worst economy possible, and always results in loss in many ways. Health is sacrificed, and a large part of the receipts from sales is paid out to doctors and for medicines.

We do not profess to be so accomplished a cook as Soyer, but there are some things we do know. One is, that neither flesh, fowl, or fish should ever be fried, when first cooked. The reason for adding the last three words, is to "save our hash," which it would be found difficult to broil on the ordinary gridiron.

All meats should be either broiled or baked. Beef should be cooked "rare," mutton, veal, and fish, and *pork*—for those who eat it—should be thoroughly cooked. In baking or roasting meats of any kind, poultry or fish, not one drop of water should be put in the pan. It destroys the flavor. Instead of water put in a little suet or lard. A piece of beef weighing ten pounds will cook well enough in one hour. If cooked till thoroughly done, the rich juices evaporate and what remains will be of little value. Rare beef is the most nutritious and the most wholesome. It is very palatable without being cooked at all. Beans are an excellent food when properly cooked. They should be boiled until thoroughly dissolved, like a paste, and then put into a dish with a small piece of butter, and baked. No one who tries this plan will ever return to the old method of baking beans with pork. We do not often lunch at a restaurant, because shortly after the operation we feel as if a sharp stick, a little longer than the diameter of the stomach, had became lodged in that organ.

Walking down town a few days since, and seeing our own name over the door of a restaurant, we ventured in and ordered " mush and milk." The milk seemed good enough, but the mush was a curiosity in its way. It consisted of numerous conglomerations of wet and dry Indian meal, each about the size of a bullet. The outer coating tasted like half cooked mush, and the inside was raw meal. The cook that made that mush might realize a fortune from the manufacture of medicated capsules. So far as our experience goes, we believe this is a fair sample of the best New York restaurants.

